# Surgical repair of an aortoesophageal fistula after salvage thoracic endovascular aortic repair: a case report

**DOI:** 10.1186/s13256-024-04605-0

**Published:** 2024-06-21

**Authors:** Hisashi Uemura, Hajime Matsue, Yasuo Suehiro, Takaya Nakagawa, Ayaka Satoh, Yoshio Teshima, Masashi Bungo, Hisashi Satoh

**Affiliations:** 1 Department of Cardiovascular Surgery, Higashi Takarazuka Satoh Hospital, Nagao-cho 2-1, Takarazuka, Hyogo Japan; 2 Department of Cardiovascular Surgery, Hyogo Medical University, Hyogo, Japan

**Keywords:** Rupture of a thoracic aortic aneurysm, TEVAR, Aortoesophageal fistula

## Abstract

**Background:**

An aortoesophageal fistula can prove to be fatal. Salvage thoracic endovascular aortic repair as a bridging therapy and radical surgery with thoracotomy should be considered while treating aortoesophageal fistula without spontaneous closure. Moreover, it is essential to select a technique that reduces the risk of reinfection. Here we report a rare case of a ruptured thoracic aortic aneurysm related to esophageal perforation by a fish bone that led to massive hematemesis and shock, and the surgical treatment of an aortoesophageal fistula that developed after salvage thoracic endovascular aortic repair.

**Case presentation:**

A 70-year-old Japanese female patient was admitted with hematemesis, thoracic pain, and shock related to esophageal perforation of a ruptured descending aortic aneurysm caused by fish bone aspiration and esophageal perforation 1 month previously. An emergency thoracic endovascular aortic repair was performed. Postoperatively, an aortoesophageal fistula that remained open and a food intake-related increase in the inflammatory response was noted. Radical blood-vessel prosthesis implantation and fistula closure were performed. The patient’s postoperative course was favorable and the patient was discharged 22 days after the blood vessel prosthesis implantation.

**Conclusion:**

Such a case of rupture of a descending aortic aneurysm related to perforation by a fish bone and an aortoesophageal fistula is considerably rare. Thus, we report the therapeutic strategy of this particular case and review the relevant literature.

## Background

Aortoesophageal fistula (AEF) was first reported in 1818 [1] and can prove to be fatal. Primary etiological factors include ruptured aortic aneurysms, foreign body ingestion, and advanced malignant esophageal tumors. Additionally, it may develop as a complication of blood vessel prosthesis implantation for aneurysms or thoracic endovascular aortic repair (TEVAR) [2, 3].

Snyder and Crawford [4] described the first patient to survive after surgery in 1983; however, few survivors have been reported thereafter. The late survival rate after conservative treatment is low; however, even with surgery, there are low survival rates and poor prognoses. Hence, emergency surgery is required when ruptured aortic aneurysms result in massive hematemesis, and treatment results and prognosis seem to be poor. Furthermore, reports have indicated the usefulness of a two-stage repair involving TEVAR as a bridging therapy [5].

Herein, we report a rare case of a ruptured thoracic aortic aneurysm related to esophageal perforation by a fish bone that led to massive hematemesis and shock, and the surgical treatment of an AEF that developed after salvage TEVAR.

## Case presentation

A 70-year-old Japanese female patient was transported to the hospital by ambulance. Upper gastrointestinal endoscopy revealed a protruding pulsatile lesion in the middle esophagus and bleeding points. Contrast-enhanced computed tomography (CT) led to the diagnosis of esophageal perforation of a thoracic aortic aneurysm. The patient was referred to our hospital for further surgery.

The patient had no relevant medical or family history. However, her appetite had markedly decreased after a fish (sea bream) bone aspiration approximately 1 month previously, leading to notable weight loss. The patient’s height and body weight were 155 cm and 38 kg, respectively. On arrival, the level of consciousness on the Glasgow Coma Scale was 11 (E3V3M5). Blood pressure and heart rate were 56/28 mmHg and 90 beats/minute, respectively, without abnormalities in the cardiac or respiratory sounds. An inflammatory response [white blood cells (WBCs), 14,400/uL] and anemia (hemoglobin, 7.0 g/dL) were observed without abnormal findings on chest radiography or electrocardiography/ultrasound cardiography.

**Fig. 1:**
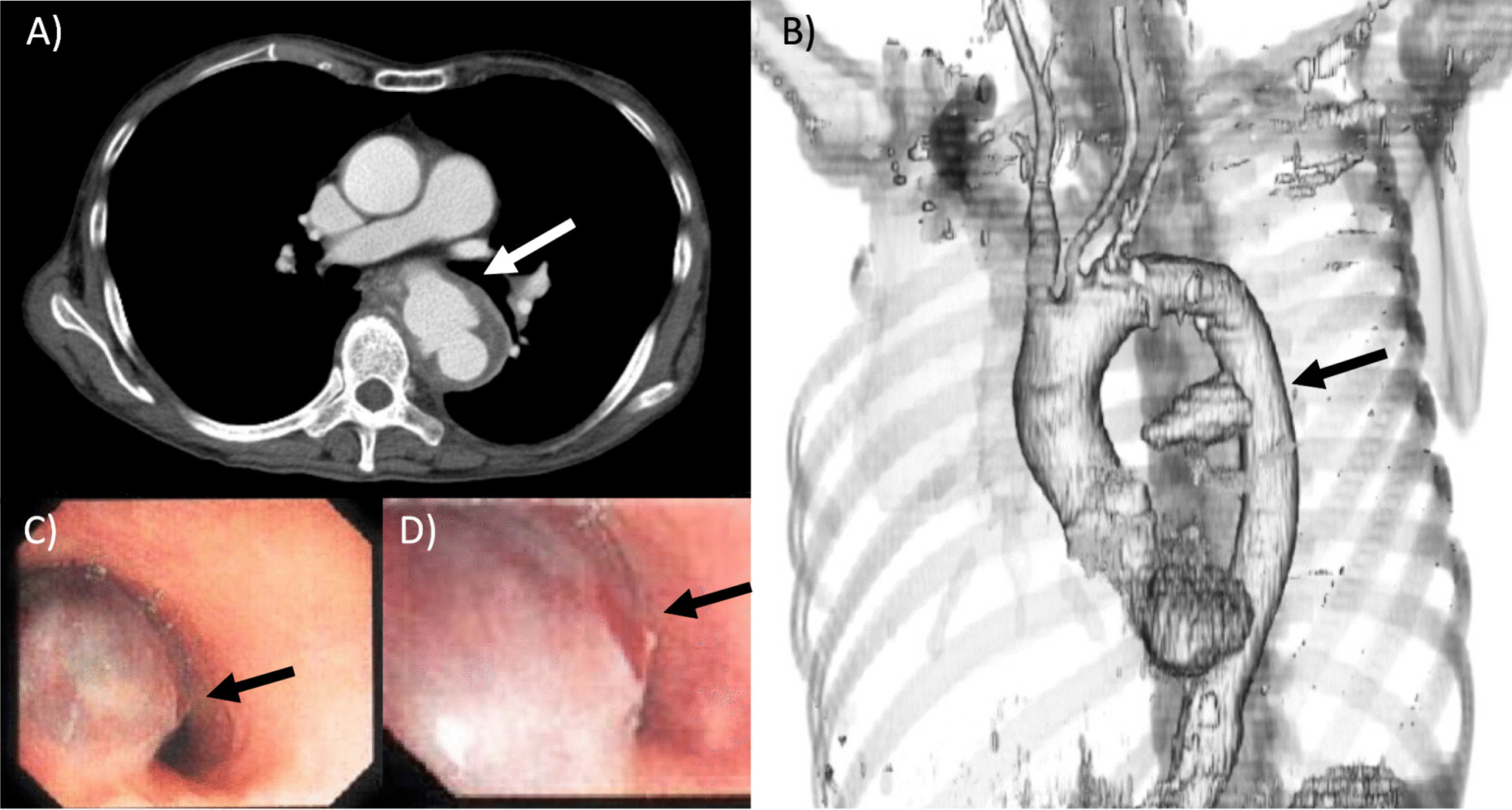
Preoperative computed tomography and endoscopy findings. **a** A computed tomography image of the rupture of a thoracic aortic aneurysm. The arrow indicates the position of the aneurysm. **b** A 3-dimensional computed tomography image. A saccular aneurysm protrudes into the descending aorta (arrow). **c** Upper gastrointestinal endoscopy. The arrow indicates aneurysm-related compression of the esophageal lumen. **d** Magnification of the image in (**c**). Site of perforation and blood points observed from the esophageal lumen (arrow)

Contrast-enhanced CT revealed a saccular descending thoracic aortic aneurysm 65 mm in diameter protruding into the esophagus (46 mm in diameter in the saccular portion; Fig. 1a, b). Horizontal cross-sections showed adventitia disturbance, suggesting rupture (Fig. 1a). Upper gastrointestinal endoscopy revealed a dark red lesion with pulsating blood points protruding into the esophageal lumen (Fig. 1c, d), suggesting that esophageal perforation related to fishbone aspiration resulted in a saccular descending aortic aneurysm, leading to aortic aneurysmal rupture and esophageal perforation.

**Fig. 2:**
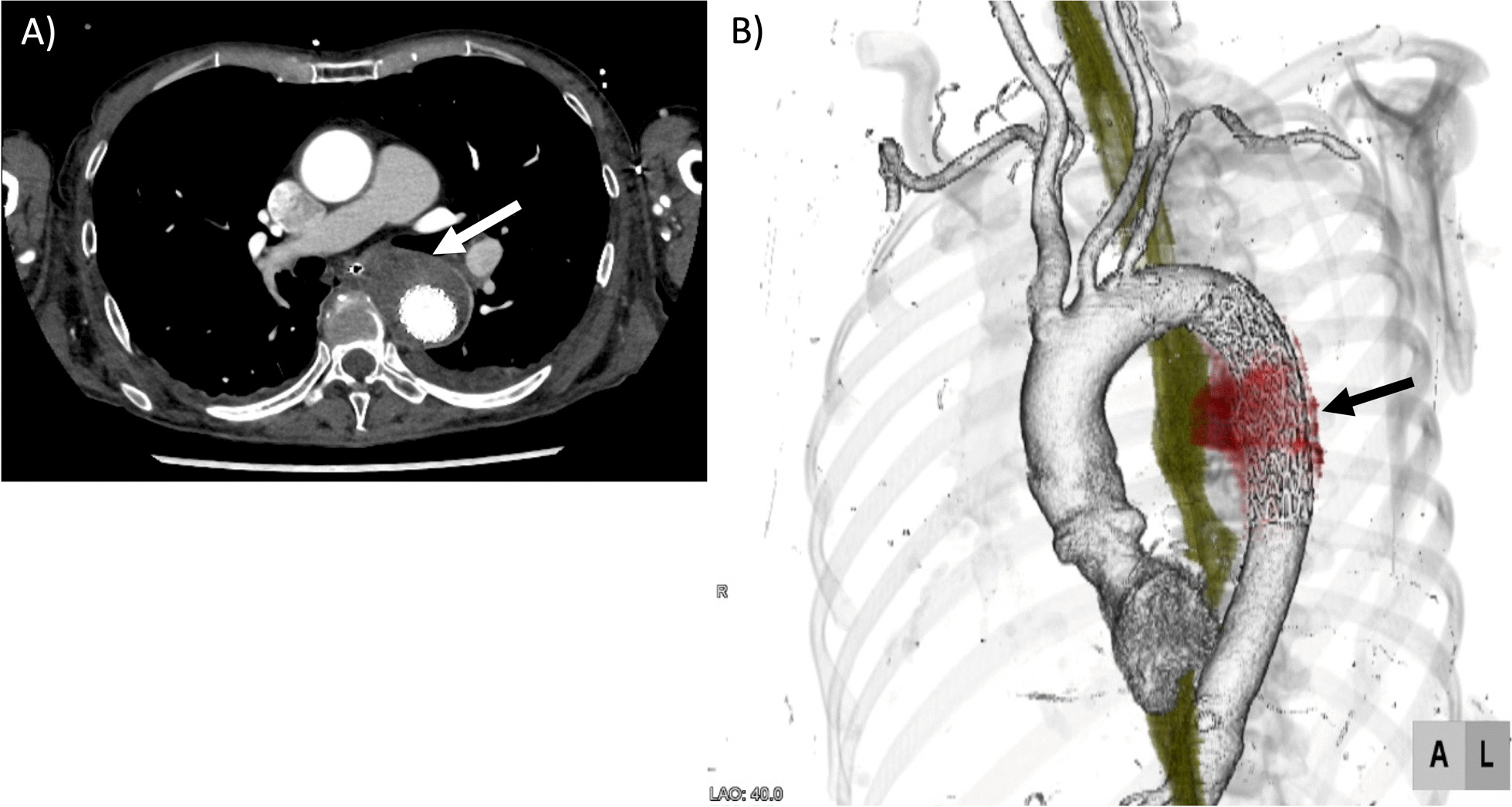
Computed tomography images after thoracic endovascular aortic repair (1 week after surgery). **a** An axial image after thoracic endovascular aortic repair. This shows that the aneurysm is thrombosed and there is no fistula formation (arrow). **b** A three-dimensional computed tomography image after thoracic endovascular aortic repair. The saccular descending aortic aneurysm is thrombosed. This shows that the stent-graft is deployed, and the aneurysm is thrombosed (arrow)

Owing to deteriorating consciousness and shock, emergency TEVAR was performed. Using a right femoral artery approach, a stent graft measuring 28 mm in diameter and 10 cm in length (Gore TAG device; WL Gore & Associates, Flagstaff, AZ, USA) was inserted into the descending aorta (zones 4–Th7). The operative time was 22 minutes, the duration of X-ray irradiation was 7 minutes, the radiation dose was 42 mGy, and the contrast medium volume was 40 mL. Extubation was performed 1 day after the surgery and contrast-enhanced CT indicated a thrombosed aneurysm without an endoleak. There were no issues regarding the insertion position (Fig. 2a, b).

**Fig. 3:**
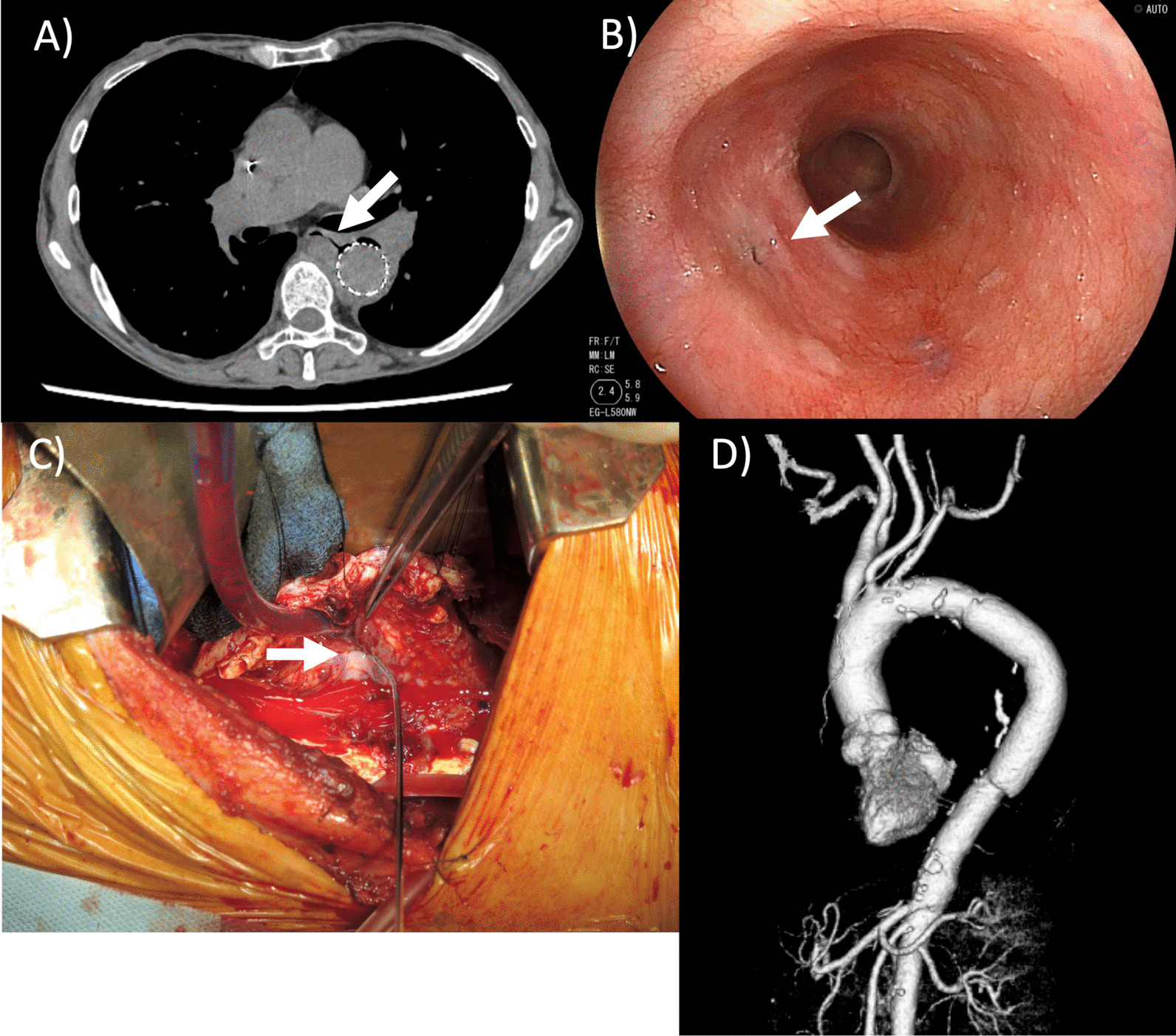
Postoperative computed tomography, endoscopy findings, intraoperative images, and postoperative computed tomography. **a** Computed tomography 2 weeks after thoracic endovascular aortic repair reveals air involving the esophagus to the periphery of the intra-aneurysmal stent-graft, suggesting the formation of an aortoesophageal fistula. **b** Upper gastrointestinal endoscopy 4 weeks after thoracic endovascular aortic repair shows that aneurysm-related compression of the esophageal lumen is relieved, but confirms an aortoesophageal fistula (arrow). **c** A fistula is confirmed in the lumen of the descending aorta. A 2-mm probe is passed through (arrow). **d** A three-dimensional computed tomography image after graft replacement. A blood vessel prosthesis is placed at a specific distance from the site of an aortoesophageal fistula

After surgery, the general condition of the patient stabilized; however, an increased inflammatory response was observed after water intake initiation. CT carried out 2 weeks after surgery revealed air involvement in the esophagus at the periphery of the intra-aneurysmal stent graft, suggesting an AEF formation (Fig. 3a). The patient was followed-up without meals. A less severe inflammatory response was noted (WBCs, 5,700/uL; C-reactive protein, 1.0 mg/dL). However, endoscopy revealed that the AEF lesion persisted (Fig. 3b). Therefore, we performed mediastinal drainage under anterior lateral thoracotomy (the fourth intercostal space) 28 days after TEVAR to wait for spontaneous AEF lesion closure. Nevertheless, the fistula became refractory and closure was unconfirmed on CT.

Esophagography performed 90 days after surgery confirmed no contrast medium leakage into the fistula. Water intake was initiated 91 days after surgery. Since there was no fever, food intake was initiated 93 days after surgery. However, fever and an increased inflammatory response were noted 95 days after surgery, the AEF persisted, and the stent graft was severely infected. Therefore, we performed the definitive AEF treatment: descending aorta replacement and entire stent-graft removal, followed by esophageal fistula repair 98 days after TEVAR.

In the right semi-recumbent position, an approach involving posterior lateral thoracotomy at the sixth intercostal space was adopted. The descending aorta strongly adhered to the esophagus and lungs with no pus; however, a small volume of pleural effusion was observed. The stent graft was palpable at both the center and the periphery. Taping was conducted for the center at the periphery of the left subclavian artery origin and the periphery of the descending aorta, approximately 4 cm peripheral to the palpable lower end of the stent graft. After systemic heparinization, a partial bypass was performed through the right femoral artery and vein. After clamping the descending aorta proximally and distally, the descending aorta, including the aneurysm lesion, was incised longitudinally. The stent-graft was completely excised.

The descending aortic wall was markedly thickened with fibrin and white moss adhering to the inner side of the aorta. The esophageal fistula was very small (2–3 mm in diameter) without any pus discharge, allowing smooth passage of a small probe (Fig. 3c). However, the esophageal adhesion was strong, making it challenging to dissect the esophagus from the surrounding tissue. Subsequently, the fistula was closed directly (4–0 Prolene) from the inner lumen of the aneurysm. The descending thoracic aorta replacement was conducted using the prosthetic graft (J Graft 24 mm, Japan Lifeline Co, Ltd., Tokyo, Japan) soaked with crystal violet, which was placed separately from the AEF. Weaning of the partial bypass was smooth, and the complete surgical procedure ended uneventfully (Fig. 3d). The surgery was completed after withdrawal from the heart–lung machine and wound closure. The entire operative time was 302 minutes. The duration of the cardiopulmonary bypass was 126 minutes.

The patient’s postoperative course was favorable. CT was performed 7 days after surgery to confirm fistula closure and food intake was initiated. The patient was discharged 22 days after the blood vessel prosthesis implantation. No aorta-associated events or digestive tract-associated complications were observed during the 1-year postoperative follow-up period.

## Discussion and conclusion

An AEF is a rare and life-threatening condition and develops as a complication of blood vessel prosthesis implantation for aneurysms or TEVAR [2, 3]. Primary etiological factors include ruptured aortic aneurysms, foreign body ingestion, and advanced malignant esophageal tumors. Esophageal and aortic factors are also involved in AEF onset. However, conditions in which esophageal perforation initially occurs and induces a thoracic aortic aneurysm are extremely rare. The etiological factors for esophageal perforation are iatrogenic in 59%, idiopathic in 15%, foreign bodies in 12%, and trauma in 9% of patients [6]. Perforation of the esophagus by a dissecting aortic aneurysm is rare, reportedly occurring in 1.5% of all cases of dissecting aortic aneurysms in Japan and in 12% in the USA and Europe, but its prevalence is thought to be increasing in recent years with the increase in diseases, such as hypertension and dyslipidemia [7].

To our knowledge, no large-scale studies have been conducted on AEF to date. Furthermore, the survival rate after conservative or surgical treatment is low and the prognosis is poor; however, the two-stage repair involving TEVAR as bridging therapy is useful [5]. To control fatal hemorrhage and prevent subsequent infectious complications, accurate treatment of aortic hemorrhage and methods to inhibit serial contamination from a fistula to the aortic wall and mediastinum are needed. Reports have indicated the usefulness of endovascular treatment for AEF under highly emergent lifesaving conditions, such as low invasiveness and short operative time [8]. Furthermore, spontaneous AEF closure can be achieved during strict follow-up of the esophagus using gastrointestinal endoscopy after TEVAR, facilitating esophageal preservation [9].

Esophageal surgery is performed in most patients; however, the details of mild cases with esophageal preservation remain controversial. Here, the AEF was initially small on endoscopy after successful endovascular treatment, and spontaneous closure was expected to facilitate esophageal preservation. However, it could not be achieved 3 months after the first TEVAR session and an increased inflammatory response related to food intake was demonstrated. Thus, blood vessel prosthesis implantation and direct closure of the AEF were performed.

AEF severity varies, and to our knowledge, no long-term follow-up survey has been conducted. Therefore, when expecting spontaneous fistula closure, there are limitations on an accurate conclusion regarding the fistula size or period required until spontaneous closure. However, as our patient had a favorable subsequent course, endovascular treatment may primarily play a role in lifesaving and bridging therapy, and a strategy to perform radical esophageal/aortic surgery after recovery may be appropriate. Radical surgical techniques include esophagectomy, gastric tube reconstruction, blood vessel prosthesis implantation, and omental packing. However, it is important to establish a specific distance between an infection-suspected site and the blood vessel prosthesis and prevent the digestive tract from being adjacent to the blood vessel prosthesis. In our case, a route was designed to separate the reconstructed artificial blood vessel from the original AEF position, and radical surgery by direct closure of the inner blood vessel wall prevented esophageal stenosis, depending on the fistula size, and avoided the postoperative vascular graft infection risks.

We encountered a patient with an AEF after salvage treatment for an esophageal perforation associated with a ruptured thoracic aortic aneurysm related to esophageal perforation by a fish bone. Salvage TEVAR as a bridging therapy and radical surgery with thoracotomy revealed favorable outcomes in treating AEF without spontaneous closure. Therefore, selecting a technique that reduces reinfection risks is necessary.

## Data Availability

The datasets generated and/or analyzed during the current study are not publicly available to protect patients’ personal information but are available from the corresponding author upon reasonable request.

## Abbreviations

AEF: Aortoesophageal fistula
CT: Computed tomography
TEVAR: Thoracic endovascular aortic repair
WBCs: White blood cells
